# Potential for Targeting Myeloid Cells in Controlling CNS Inflammation

**DOI:** 10.3389/fimmu.2020.571897

**Published:** 2020-10-06

**Authors:** Igal Ifergan, Stephen D. Miller

**Affiliations:** ^1^Department of Microbiology-Immunology, Feinberg School of Medicine, Northwestern University, Chicago, IL, United States; ^2^Interdepartmental Immunobiology Center, Feinberg School of Medicine, Northwestern University, Chicago, IL, United States

**Keywords:** myeloid cels, multiple sclerosis, chemokines, GM-CSF, miRNAs, nanoparticles, M-CSF

## Abstract

Multiple Sclerosis (MS) is characterized by immune cell infiltration to the central nervous system (CNS) as well as loss of myelin. Characterization of the cells in lesions of MS patients revealed an important accumulation of myeloid cells such as macrophages and dendritic cells (DCs). Data from the experimental autoimmune encephalomyelitis (EAE) model of MS supports the importance of peripheral myeloid cells in the disease pathology. However, the majority of MS therapies focus on lymphocytes. As we will discuss in this review, multiple strategies are now in place to target myeloid cells in clinical trials. These strategies have emerged from data in both human and mouse studies. We discuss strategies targeting myeloid cell migration, growth factors and cytokines, biological functions (with a focus on miRNAs), and immunological activities (with a focus on nanoparticles).

## Introduction

Myeloid cells play critical roles in the health and diseases of the central nervous system (CNS). For example, myeloid cells constitute a significant proportion of the cells found within perivascular infiltrates in CNS lesions of Multiple Sclerosis (MS) and its animal model, experimental autoimmune encephalomyelitis (EAE) (1–3). Myeloid cells are also critically involved in the secondary damage in spinal cord injury (SCI) and traumatic brain injury (TBI) (4–6). These myeloid cells have the ability to attract other immune cells, release neurotoxic factors, phagocytose proteins and debris and promote the expansion, and polarization of antigen-specific T cells in the CNS. In addition to their capacity to induce and sustain inflammation, myeloid cells are also critically involved in communication with glial cells and neurons, as well as in promoting and maintaining peripheral tolerance (7–9).

MS is an inflammatory autoimmune disease wherein cells of the immune system initiate an attack against myelin in the CNS that supports axonal conduction. The immune response in MS is thought to be mediated by autoreactive T lymphocytes that recognize myelin peptides. Typically, demyelination is associated with an accumulation of T lymphocytes (lymphoid component of infiltrates) and monocytes/ macrophages/ dendritic cells (myeloid cells component of infiltrates) that arise from the migration of peripheral blood immune cells across the CNS microvascular endothelium (10–12).As they infiltrate the CNS, encephalitogenic T lymphocytes require the presence of these blood-derived antigen-presenting cells (APCs) to further sustain lymphocyte proliferation and cytokine polarization in the CNS compartment (13–16). The role of these peripherally-derived myeloid cells in CNS inflammation will be the focus of the present review.

## Role of Myeloid Cells in the Pathogenesis of MS and Other Autoimmune Diseases

Experimental autoimmune encephalomyelitis is a commonly utilized mouse model of MS that recapitulates many aspects of the human disease such as the CNS inflammation, encephalitogenic T cell infiltration, and attack of oligodendrocytes resulting in demyelination. Although not perfect, EAE has allowed uncovering some of the molecular pathways governing the pathogenesis of MS such as elucidating the pathogenic role of T_H_17 lymphocytes. In addition, EAE models were critical in identifying and testing new therapeutic agents such as glatiramer acetate (GA) and Natalizumab (17).

Although myeloid APCs play a prominent role in the pathogenesis of MS, there has been little consideration given to targeting these cells as an MS therapy. Some of the current MS disease-modifying therapies may act on myeloid cells even if these cells were not the original intended targets (18). However, interfering directly with myeloid cell has proven to be efficacious in other diseases including psoriasis with multiple drugs targeting IL-23 (Guselkumab, Risankizumab, and Tildrakizumab) or IL-12 and IL-23 (Ustekinumab) (19), Crohn's disease and ulcerative colitis targeting IL-12 and IL-23 (Ustekinumab) (20), rheumatoid arthritis targeting IL-1 (Anakinra) (21), systemic juvenile idiopathic arthritis targeting IL-1β (Canakinumab) (22), and many others. There are ongoing clinical trials in rheumatoid arthritis, stroke, atherosclerosis, and cancer using agents that target myeloid cells and their products. Biber et al. have provided a recent comprehensive review of drugs in clinical trials targeting myeloid cells in CNS diseases such as Alzheimer's disease, brain tumors, and inflammatory pain, as well as for other CNS diseases (23).

As we will discuss in this review, multiple tools have been developed in the EAE models of MS demonstrating significant regulation of disease progression by various approaches blocking myeloid cell activation and effector function, but to date, these approaches have not been tested for therapeutic efficacy in MS patients.

## Targeting Myeloid Cell Migration

The first strategy we will discuss is interference with peripheral myeloid cell migration to the CNS. The blood-brain barrier (BBB), composed of tightly bound endothelial cells (ECs), regulates the entry of blood-borne molecules and immune cells into the CNS. Under physiological conditions, a limited number of peripheral blood immune cells gain access to the CNS, a process called immune surveillance (24). During an inflammatory process, meningeal, and BBB-ECs amplify the migration of immune cells into the CNS parenchyma, in a multi-step process that involves selectins, chemokines and cell adhesion molecules (25). BBB-ECs express cell adhesion molecules such as intercellular adhesion molecule (ICAM)-1, vascular cell adhesion molecule (VCAM)-1, activated leucocyte cell adhesion molecule (ALCAM), and melanoma cell adhesion molecule (MCAM) which mediate at least in part, the adhesion process and the transmigration of leucocytes to the CNS through their interaction with integrins αLβ2 [leucocyte function-associated antigen (LFA)-1], α4β1 [very late antigen (VLA)-4], CD6, and MCAM respectively (26–31). Interfering with immune cell trafficking across the BBB by targeting adhesion molecules has proven to be beneficial in reducing clinical disease activity and pathological indices in MS (32). Indeed, Natalizumab, which blocks VLA-4, the ligand of VCAM-1, is reported to reduce migration of most leukocyte subtypes, including myeloid cells, into the brain.

More recently, a new adhesion molecule expressed by BBB-ECs called Nerve injury-induced protein (Ninjurin)-1 was described (33). Ninjurin-1 is a membrane protein known to interact in a homophilic manner through an extracellular residue-binding motif (34). On immune cells, Ninjurin-1 was weakly expressed by lymphocytes, but highly expressed by peripheral myeloid APCs including monocytes, macrophages and dendritic cells (DCs), in humans and mice. Interestingly, Ninjurin-1 was also found to be expressed in MS lesions. Ninjurin-1 neutralization specifically abrogated the adhesion and migration of human monocytes across a monolayer of BBB endothelial cells, without affecting lymphocyte recruitment. Moreover, Ninjurin-1 blockade during the course of EAE reduced infiltration of peripheral myeloid cells and reduced clinical disease activity and histopathological indices of EAE (33).

Another adhesion molecule involved in the migration of peripheral myeloid cells is junctional adhesion molecule (JAM)-like (JAML). JAMs are type I transmembrane proteins differentially expressed at the junctions of ECs, epithelial cells, and on various leukocytes (35). Similarly to Ninjurin-1, JAML can interact in a homophilic manner (36). It was observed that JAML is expressed by BBB-ECs, and has an increase expression in MS lesions compared to normal appearing white matter (37). In addition, human monocytes and CD8^+^ T cells were found to express JAML, and its level was significantly increased on RRMS patients when compared control subjects: 80 vs. 52% for monocytes, and 5.5 vs. 2.1% and for CD8^+^ T cells. These data reveals that JAML might be a more important adhesion molecule for monocytes than for CD8^+^ T cells. However, migratory capacity of both cell types was significantly compromised when JAML was blocked.

Chemotactic cytokines (chemokines) are secreted proteins that regulate the migration of leukocytes. Chemokine receptor signaling plays a central role in cell migration during inflammatory responses in autoimmune and infectious diseases as well as in cancer. There are ~50 chemokines and 20 receptors known at this time. Blockade of CCR1 and CCR2 have been the two majors targets in a half dozen MS clinical trials (38). The chemokines CCL3 (macrophage inflammatory protein-1α–MIP-1α) and CCL5 (regulated on activation, normal T cell expressed and secreted—RANTES) bind to CCR1, while CCL2 (monocyte chemoattractant protein 1—MCP-1) binds to CCR2. Both lymphoid and myeloid cells express CCR1 and CCR2, with monocytes/macrophages/DCs the cells where these chemokine receptors are most abundant (39–42). In animal models of MS, it was shown that CCR1-deficient animals developed a less severe disease (43), while CCR2-deficient mice were completely resistant to disease induction (44, 45), highlighting the importance of signaling through these chemokine receptors for disease initiation. In addition, it has been shown that CCR2^+^Ly-6C^hi^ monocytes are rapidly recruited to the inflamed CNS in EAE and are crucial for the effector phase of disease. Selective depletion of this specific monocyte subpopulation through engagement of CCR2 significantly reduced disease severity (46). CCR1^+^ and CCR2^+^ macrophages were both found in active MS lesions (47, 48). The role of chemokines and their receptors are now well-characterized in MS and other inflammatory diseases. The potential for a therapy targeting this signaling pathway is well-recognized. However, none of the chemokine-directed MS clinical trials has shown robust clinical efficacy. Similar lack of clinical responses have also been reported in therapeutic trials targeting chemokines in other diseases such as rheumatoid arthritis, psoriasis, asthma, and many others (49). The issue may lie in the redundancy of chemokine/chemokine receptor action, in which case, it may be beneficial to develop strategies employing multiple antagonists simultaneously.

As innate cells, myeloid cells express pattern recognition receptors (PRRs). PRRs include Toll-like receptors (TLRs), RIG-I-like receptors, NOD-like receptors, and C-type lectin receptors (CLRs) (50). Selectins, which are part of the C-type lectins family, are known to play a crucial role in the control of leukocyte trafficking and homing to sites of inflammation (51). Selectins are particularly important for the rolling of cells on endothelial cells, an important component of migration of myeloid cells into tissue sites of inflammation (52). More recently, it was uncovered that CLEC12A, a CLR, was involved in facilitating binding and transmigration of DCs across the BBB in response to CCL2 chemotaxis (53). In EAE, CLEC12A^−/−^ mice displayed delayed disease onset and significantly reduced disease severity. Additionally, in a chronic model of EAE, anti-CLEC12A antibody treatment initiated at disease initiation also delayed onset and lessened disease severity. Anti-CLEC12A antibody administration to mice undergoing relapsing-remitting EAE after disease onset, resulted in less severe disease relapse (53). Although the ligand of CLEC12A is currently unknown, it was suggested that the ligand is present on BBB endothelial cells (53).

## Targeting Myeloid Cell Activation and Cytokines

### Growth Factors

Abundance of immune cells as well as cytokines, chemokines and immunoglobulins in MS plaques and their accumulation in the cerebrospinal fluid (CSF) of MS patients, support the notion that MS is an inflammatory disorder. These observations lend support to the idea that immune cell products, especially cytokines, have an important role in both the induction and progression of MS. Targeting cytokines has been a successful strategy used in therapy of other inflammatory diseases. For example, blockade of tumor necrosis factor (TNF) has shown positive results in Rheumatoid Arthritis and Crohn's disease (54, 55). As of December 2016, TNF inhibitors were the world's leading drug class, with sales of more than US $30 billion and used in more than seven million patients (56). In MS, the first treatment approved for RRMS was interferon (IFN)-β, thus showing that cytokines manipulation is potentially a good strategy.

Current data suggests that MS, and its animal model, EAE, are driven by both T_H_1 lymphocytes, producing IFN-γ, interleukin (IL)-2 and TNF, and T_H_17 lymphocytes, producing IL-17, IL-21, IL-22, and Granulocyte-macrophage colony-stimulating factor (GM-CSF also known as CSF-2). Surprisingly, IFN-γ, IL-12, IL-17A, IL-17F, IL-21, and IL-22 have all been shown to be dispensable for the development of EAE [reviewed in (57); discussed here (58)]. However, in 2011, the CNS pathogenicity of T_H_17 cells was reported to be primarily associated with their production of GM-CSF (59, 60). GM-CSF production by T cells has been correlated with pathogenesis in several autoimmune diseases, including MS, rheumatoid arthritis, and myocarditis. It was reported that IL-1β- and IL-23-induced production of GM-CSF by CNS-infiltrating CD4^+^ T cells is essential for the induction of EAE (59, 60).

GM-CSF is a hematopoietic growth factor produced by a number of hematopoietic and non-hematopoietic cell types including activated CD4^+^ T cells, monocytes/macrophages, B cells, NK cells, endothelial cells and epithelial cells. GM-CSF has a wide array of functions, notably the survival and activation of myeloid cells, the ability to induce differentiation of dendritic cells (DCs), the polarization of macrophages toward a pro-inflammatory M1 phenotype, enhanced antigen presentation, the induction of complement- and antibody-mediated phagocytosis, and the mobilization of monocytes and other myeloid populations from bone marrow to blood (61–63).

The GM-CSF receptor (GM-CSF Rc) is a heterodimer comprised of a specific low-affinity α chain (CD116; GM-CSF Rα) and a common β chain (CD131; GM-CSF Rβ) that is shared by IL-3 and IL-5 (64). The GM-CSF Rc is expressed in multipotent myeloid progenitor cells and continues to be expressed throughout myeloid development on monocytes, DCs, macrophages and neutrophils (65–67). It is not expressed by T and B lymphocytes (67). Thus, most of the suspected direct effects of the GM-CSF in diseases are focused on myeloid cells (peripheral and CNS resident).

Findings related to the function of GM-CSF signaling in EAE pathology have been recently reviewed (68). In brief, in EAE, GM-CSF is necessary for disease as GM-CSF KOs were found to be resistant to disease induction (69). Disease can be rescued by the administration of recombinant GM-CSF. Adoptive transfer using cytokine-deficient mice showed that wild-type, IL-17A^−/−^, and IFNγ^−/−^ T cells induced EAE with similar kinetics. By contrast, GM-CSF^−/−^ T cells were incapable of inducing EAE and invading the CNS (59). Due to the variety of cells GM-CSF can stimulate, it became important to determine the cell population in which signaling was necessary for disease. A bone marrow chimera study determined that peripheral myeloid cells, but not microglia, are key responders (59). This corresponds with earlier observations that GM-CSF administration stimulated CD11b^+^ Ly6C^hi^ inflammatory monocytes into the circulation (70). Circulating Ly6C^hi^ monocytes traffic across the blood-brain barrier, up-regulate pro-inflammatory molecules, and differentiate into central nervous system DCs and macrophages (70). These data were confirmed recently using conditional gene targeting in which the β chain of the GM-CSF receptor (*Csf2rb*) was deleted in specific subpopulations throughout the myeloid lineages (71). It was found that deletion of *Csf2rb* in CCR2^+^Ly6C^hi^ monocytes phenocopied the EAE resistance seen in complete *Csf2rb*-deficient mice.

In humans, GM-CSF levels in the CSF are higher in patients with active MS than in patients in remission (72). Also, untreated MS patients had significantly greater numbers of CD4^+^ GM-CSF^+^ T cells and CD8^+^ GM-CSF^+^ T cells in peripheral blood compared with healthy controls and with IFN-β-treated MS patients (73). In addition, IFN-β significantly suppressed GM-CSF production by T cells *in vitro*. More recently, the Canadian B cells in MS Team uncovered a subset of memory B cells producing GM-CSF (74). *In vitro*, GM-CSF–expressing B cells efficiently activated myeloid cells in a GM-CSF–dependent manner, and *in vivo*, B cell depletion therapy resulted in a GM-CSF–dependent decrease in pro-inflammatory myeloid responses of MS patients.

In light of the critical role of GM-CSF in the pathogenesis of MS and other inflammatory diseases, multiple tools have been developed targeting either the cytokine or the receptor. First tested in EAE, it has been shown that blocking antibodies against GM-CSF in chronic (C)-EAE (69) or antibodies against GM-CSF Rα in C-EAE and Relapsing-Remitting (RR)-EAE (75) were able to prevent disease if given at the time of EAE induction (day 0). Mice treated with anti-GM-CSF after disease onset completely recovered within 20 days of treatment in a model of C-EAE (69). Therapeutic treatment with anti-GM-CSF Rα ameliorated progression of C-EAE and resulted in a significant reduction of the relapse severity of RR-EAE (75). Blockade of the GM-CSF Rα led to a reduction of activated mDCs, and reduced pro-inflammatory cytokine production by CD11b^+^ Ly6C^+^ inflammatory monocytes. Additionally, anti-GM-CSF Rα altered the expression of chemokine receptors, leading to the possibility that antibody treatment may impede cell migration (75).

Logically, the next step is to test the therapeutic potential of GM-CSF targeting in humans. A review of tools developed for clinical trials can be found here (76). At this time, GM-CSF blocking antibodies have been tested in Rheumatoid Arthritis and have shown promising results. As for MS, only one drug has been tested in clinical trials: MOR-103 (also known as GSK3196165 or otilimab), a human antibody to GM-CSF. The results of a Phase Ib clinical trial employing MOR-103 in patients with relapsing-remitting or secondary-progressive MS have shown the drug to be safe and well-tolerated, although with modest efficacy (77). At this moment, there are no ongoing clinical trials targeting GM-CSF or GM-CSF receptor in MS.

Another important growth factor regulating myeloid cell function is macrophage colony-stimulating factor (M-CSF also known as CSF-1). M-CSF is ubiquitously produced in the steady state by a variety of cells, including endothelial cells, fibroblasts, osteoblasts, smooth muscle, and macrophages, and can be detected in plasma at ~10 ng/ml (78–80). The levels of circulating M-CSF are upregulated in pregnancy (81) as well as in many different pathologies including cancer, autoimmune diseases and chronic inflammation (82–86). M-CSF stimulates progenitor cells from bone marrow and plays an important regulatory role in the survival, proliferation (in mice), differentiation, phagocytosis, and chemotaxis of myeloid cells, including monocytes, macrophages, DCs, and microglia (87–89). The effects of M-CSF are mediated by signaling through the type III tyrosine kinase transmembrane receptor CSF-1R (CD115), which is encoded by the c-*fms* proto-oncogene (90). IL-34 is also able to bind CSF-1R with similar outcomes as to M-CSF binding (88). However, M-CSF and IL-34 present differences in their spatiotemporal expression patterns, and thus seem to play complementary roles in their biological activities on target cells (88, 91, 92). CSF-1R is expressed by myeloid cells such as monocytes, macrophages, DCs, and microglia, as well as by trophoblasts, neural progenitor cells and epithelial cells (93, 94).

There is ongoing debate about whether M-CSF is a pro-inflammatory or pro-repair cytokine. M-CSF seems to be essential for the survival and renewal of tissue-resident macrophages, but not for circulating myeloid cells. Indeed, in the osteopetrotic Csf1^op^/Csf1^op^ mouse, which harbor an inactivating mutation in the coding region of the *CSF-1* gene and are M-CSF deficient, the functions and numbers of several tissue macrophage populations are altered while there is no difference in monocyte populations in the blood (95). These findings were later confirmed in mice deficient for a specific enhancer for *Csf-1r* gene, the fms-intronic regulatory element (FIRE) (96). Csf1r^Δ*FIRE*/Δ*FIRE*^ mice present a deficit in tissue resident macrophages in the brain (microglia), skin, kidney, peritoneal, and heart without significant differences in blood monocytes. During inflammation, the presence of monocytes in inflamed tissue is critical for proper immune responses, notably due to their capacity to traffic to draining lymph nodes and their ability to present antigens to T cells (2, 97–103). While tissue resident macrophages also participate in inflammatory processes, their role in promoting tissue repair and regeneration is critical (104, 105). For example, M-CSF favors kidney and liver repair after acute injury (106–108). Moreover, M-CSF is used to drive human and in mouse macrophage differentiation *in vitro* into an anti-inflammatory (M2) phenotype (109–111). In EAE, it was shown that peritoneal APCs treated with M-CSF and pulsed with MOG_35−55_, the disease initiating peptide, were able to suppress ongoing EAE when injected at the time of disease initiation or significantly reduce the severity of the disease when injected at day 7 post-immunization (112). These M-CSF activated APCs were demonstrated to induce a Treg profile from CD4^+^ T cells (CD25^+^ FoxP3^+^) with increased secretion of IL-10 and decreased secretion of IL-17, IFN-γ, and TNF (112).

However, as mentioned earlier, elevated levels of M-CSF are also observed in different pathologies. There are multiple publications linking M-CSF/IL-34 and CSF-1R signaling in models of arthritis (113–116), diabetes (117), systemic lupus erythematosus (85, 118), cancer (119–121), amyotrophic lateral sclerosis (122), Parkinson's disease (123), and Alzheimer's disease (124–126). In an effort to determine the role of M-CSF/IL-34 and CSF-1R signaling in MS, different groups used potent c-fms tyrosine kinase inhibitors, which block M-CSF signaling. Ki20227 (127), imatinib (128), GW2580 (128, 129), sorafenid (128), and PLX5622 (130) are all tyrosine kinase inhibitors that have shown to effectively treat C-EAE. GW2580 has the greatest apparent specificity for CSF-1R vs. the other kinase inhibitors (131). Amelioration of EAE using Ki20227 was associated with the suppression of myeloid cell expansion in the spleen and reduction in MOG-specific T-cell proliferation (127). GW2580 and sorafenib suppressed TNF-α production by macrophages whereas imatinib and sorafenib both abrogated PDGF-induced proliferation of astrocytes (128). PLX5622 effect was associated with microglia and macrophage ablation from the white matter (130). However, in the cuprizone model of CNS demyelination, which allows study of the remyelination process with little involvement of the peripheral immune cells (132), injection of M-CSF reduced demyelination by boosting microglia activity (133). Tamoxifen-induced conditional deletion of the CSF-1R in microglia from cuprizone-fed mice caused aberrant myelin debris accumulation and reduced microglial phagocytic responses (89, 133). These data indicate that M-CSF plays an important role in ability of microglia to clear myelin debris and to support proper remyelination, and suggest M-CSF functions as a critical factor in tissue repair. These divergent results exemplify the various functions of M-CSF/IL-34 and CSF-1R signaling on cells. The possible contribution of M-CSF signaling to both inflammatory and repair processes suggest that targeting M-CSF in MS may be problematic. However, although there is an increase of myeloid cells in MS lesions, the expression of CSF-1R is lower in MS lesions when compared to normal appearing white matter (134). It is thus possible to hypothesize that a therapeutic treatment targeting M-CSF in MS would primarily target peripheral myeloid cells rather than those in the CNS.

There are now multiple tools targeting M-CSF signaling approved for human therapy, especially for cancer. Imatinib was the first tyrosine kinase inhibitor approved for the treatment of chronic myelogenous leukemia (135). Imitanib is also now in clinical trials for the treatment of different pathologies, such as rheumatoid arthritis, type I diabetes and asthma, for which positive results of a phase 2 clinical trial were recently published (136). Sorafenib is approved for the treatment of primary kidney cancer and advanced primary liver cancer (137). Although there are side effects related to these inhibitors, an important advantage of tyrosine kinase inhibitors is the fact they can be administered orally to the patients. In September 2019, a phase 3 clinical trial for RRMS was started testing the efficacy of evobrutinib, a Bruton's tyrosine kinase inhibitor. Although this is not a CSF-1R inhibitor, it shows: (1) the desire to develop oral treatments in MS, and (2) the possibility of targeting tyrosine kinases in MS. Bruton's tyrosine kinase are critical for B cell receptor signaling and is also involved in TLR signaling as well as inflammasome activation in myeloid cells (138)

### Cytokines

As mentioned earlier, blockade of myeloid specific cytokines IL-1β, IL-12, and IL-23 have proven to be efficient therapies in multiple diseases such as Crohn's disease, ulcerative colitis, rheumatoid arthritis, psoriasis, and systemic juvenile idiopathic arthritis. These cytokines are all involved in CD4^+^ T lymphocytes differentiation. While IL-12 is critical for T_H_1 induction (139), IL-1β and IL-23 are both involved in T_H_17 differentiation and promote the encephalitogenic capacity of these cells by inducing GM-CSF expression (60, 140, 141). In EAE, mice lacking IL-1β, or the receptor, IL-1R, developed a milder disease than WT animals (140, 142–145). Moreover, specific ablation of IL-1R on CD4^+^ T cells resulted in significantly reduced disease severity (146), confirming the importance of IL-1β signaling on T cells to induce a full EAE. In addition, rats treated with an IL-1 receptor antagonist (IL-1Ra), which blocks the biological activity of IL-1β, developed milder signs of EAE compared to control animals (147). As IL-1β secretion is the result of inflammasome activation, mice treated with a blocking agent for the inflammasome component NLRP3 exhibited decreased EAE severity (148). In MS, it was shown that IL-1R expression is significantly higher in CD4^+^ T cells from RRMS patients than from healthy controls (149). IL-1β expression was also found to be significantly increased in MS lesions when compared to tissue from other neurological diseases (150). Interestingly, multiple treatments used in MS [e.g., IFN-β, glatiramer acetate, and natalizumab] have shown to increase IL-1Ra expression and/or to decrease IL-1β production (151, 152)]. Multiple tools have been developed to block IL-1β activity: the recombinant IL-1Ra Anakinra used for rheumatoid arthritis, the neutralizing IL-1β antibody Canakinumab used for systemic juvenile idiopathic arthritis as well as cryopyrin-associated periodic syndrome, and the soluble decoy IL-1 receptor (Rilonacept) also use for cryopyrin-associated periodic syndromes (153). At this time, Anakinra is the only IL-1β-targeting drug in clinical testing for MS. This Phase I/II clinical trial just started a few months ago, and at this time, it is still in the recruitment phase (NCT04025554).

IL-12 and IL-23 are heterodimeric cytokines that share a common subunit IL-12p40. The other subunit needed to form IL-12 is IL-12p35, while the other subunit to form IL-23 is IL-23p19. IL-12 signals through the IL-12 receptor (IL-12R) composed of the IL-12Rβ1 and IL-12Rβ2 subunits, while IL-23 signals through IL-23R and IL-12Rβ1 (154). Thus, IL-12Rβ1 is required for biological response to both IL-12 and IL-23. When specific gene ablation was tested for the different receptor chains of IL-12 and IL-23, it was found that IL-12Rβ1^−/−^ mice were completely resistant to EAE (155). However, IL-12Rβ2^−/−^ mice developed severe EAE, extensive inflammation and demyelination, and higher production of pro-inflammatory cytokines than WT animals (156). Finally, similar to IL-12Rβ1^−/−^ mice, IL-23R^−/−^ mice were completely resistant to EAE induction (157). As for the cytokines, mice deficient for the subunits IL-23p19 or IL-12p40 were resistant to EAE. By contrast, mice in which the subunit IL-12p35 was deleted were highly susceptible to EAE (158). In addition, treatment with anti-IL-12p40 antibodies inhibited both murine and primate models of EAE (159–161). Treatment with anti-IL-23p19 antibodies reduced the clinical severity and prevented relapsing EAE by inhibiting epitope spreading (162). These results led to the conclusion that IL-23 was a more critical factor than IL-12 in the inflammatory response observed in EAE. Nevertheless, there are multiple studies linking both cytokines to MS pathology. It was demonstrated that peripheral blood monocytes from progressive MS patients produced increased amounts of IL-12 compared to controls and that IL-12 production correlated with disease activity (163). Another study showed an augmented level of IL-12 mRNA-expressing cells in the peripheral blood and the CSF of MS patients when compared to controls (164). There was also elevated levels of IL-12p70 detected in plasma from MS patients compared to healthy individuals. A more recent report showed that both RRMS and secondary progressive MS patients had increased levels of IL-12p40 mRNA compared with controls during the development of active lesions (165). IL-12p40 and IL-23p19 have also been detected in human MS lesions (166, 167). Based on this and other data, there was hope that Ustekinumab, an IL-12p40 neutralizing antibody, would be efficacious for treatment of MS. However, disappointingly no clinical improvement in the treatment group compared to the placebo was found (168). Possible reasons for the failure of Ustekinumab are the broad range of MS patients in the trial, many having very severe symptoms and long-standing disease. Also, there may be weak bioavailability of the drug as Ustekinumab may be inefficient in crossing the BBB (169). At this time there are no ongoing trials targeting IL-12/IL-23 in MS despite the impressive results in the various animal models of the disease.

## Targeting Biological Functions of Myeloid Cells

MicroRNAs (miRNAs) are small non-coding RNAs of 17–25 nucleotides that regulate gene expression by inducing mRNA degradation or by interfering with translational machinery of mRNAs (170). It is predicted that more than 60% of protein-coding genes are regulated by miRNAs (171). They are key regulators of various biological processes including immune cell lineage commitment, differentiation, maturation, and maintenance of immune homeostasis and normal function [reviewed in (60)]. Extensive evidence demonstrates that miRNAs play crucial roles in the development, differentiation, and function of different immune cells, such as B and T lymphocytes, DCs and macrophages (172–175). In the last few years, miRNAs have drawn a lot of interest due to their involvement in the pathogenesis of cancer, inflammatory and autoimmune diseases [reviewed in (176)].

In MS patients, expression studies using whole blood (177), PBMCs (178), as well as brain sections (179) identified multiple deregulated miRNAs. Of these miRNAs, three were consistently upregulated across multiple studies and directly affecting myeloid cell functions: miR-223, miR-155 and miR-146a. miR-223 is induced by the myeloid transcription factors PU.1 and CCAAT/enhancer-binding protein-β (C/EBPβ) (180). miR-223 expression is mainly confined to myeloid cells and is induced during the lineage differentiation of myeloid progenitor cells. It was shown to negatively regulate both the proliferation and activation of neutrophils (181). Moreover, miR-223^−/−^ macrophages exhibited enhanced pro-inflammatory M1, but decreased regulatory M2 responses (182). It was later described that miR-223 is required for efficient M2-associated phenotype and function (183). Moreover, a low functional level of the miR-223 is essential for monocyte differentiation. In MS patients, miR-223 was found significantly increased in blood, PBMCs and active MS lesions compared with control subjects (177, 179). During EAE development, the expression level of miR-223 is dramatically increased in myeloid cell populations, but not in other cell types, and was maintained at comparable levels between disease onset and peak of disease (184). Surprisingly, although miR-223 expression is associated with M2 macrophages and microglia (183), it was shown that miR-223 KO mice present a milder course of EAE than WT mice (184–186). Reduced disease severity was also observed in adoptive transfer EAE induced by transfer WT T lymphocytes into miR-223 KO recipient mice compared to transfer into WT recipient mice, demonstrating the importance of miR-223 on the APCs side rather than on the T cells side (184). Our group demonstrated that while M1-like macrophages were upregulated in KO mice, DCs showed a reduced inflammatory profile characterized by increased PD-L1 expression and decreased expression of IL-1β, IL-6, and IL-23, all cytokines involved in differentiating and sustaining a T_H_17 profile (184). Significantly, APCs from miR-223 KO mice have a comparable ability to drive T_H_1 cells, but possess a reduced capacity to drive T_H_17 cells (184). Moreover, it was shown that monocytic-myeloid-derived suppressor cells (MO-MDSCs) isolated from miR-223^−/−^ suppressed T cell proliferation and cytokine production *in vitro* and regulated EAE more efficiently than MO-MDSCs derived from WT animals (186). The enhanced suppressive function of miR-223^−/−^ MO-MDSCs was associated with higher expression of *Arg1* and *Stat3*, which are miR-223 target genes (186). Interestingly, *Stat3* controls the expression of PD-L1 on APCs (187), consistent with the previous observation of PD-L1 upregulation on DCs in miR-223^−/−^ animals. Although these results point to miR-223 as a potential therapeutic target in MS, it is important to note that in a model of lysolecithin-induced demyelination, the absence of miR-223 was demonstrated to lead to impaired CNS remyelination and myelin debris clearance (183). The impaired capacity of M2 polarization by macrophages and microglia is likely a significant factor contributing to the decreased remyelination capacity in miR-223 KO mice. In particular, microglia adopting an M2 profile are critical for proper remyelination (188, 189). Thus, when targeting miR-223 in MS, it is important to keep in mind the different implications of such therapy.

miR-155 has drawn a lot of attention for its possible role in MS as detailed in a recent review (190). miR-155 has been shown to be upregulated in active MS lesions (179) as well as in CD14^+^ monocytes isolated from the blood of RR-MS patients compared to control donors (191). While miR-223 expression is limited to myeloid cells, multiple immune cell populations express miR-155 such as B cells, T cells, macrophages and DCs (192). miR-155 is found at low levels in both myeloid and lymphoid cells, but its expression is upregulated following cellular activation via antigen, Toll-like Receptor (TLR) ligands, and inflammatory cytokines. An important target of miR-155 is Src homology 2 (SH2)-domain containing inositol-5′-phosphatase 1 (SHIP-1) (193). SHIP-1 is an enzyme that inhibits phosphoinositide 3-kinase (PI3K) activity, which governs cellular responses to multiple stimuli, cell proliferation and cell survival (194). Thus, it is believed that miR-155 dysregulation would have critical consequences. Indeed, forced expression of miR-155 in hematopoietic stem cells by a retroviral vector leads to severe splenomegaly as well as increased myeloid cell populations in the bone marrow and in circulation (195). In addition, it has been reported that in absence of miR-155, mice displayed altered immune responses to infectious agents, due to defective functions of B cells, T cells, and DCs (196). Focusing on myeloid populations, it was shown that DCs lacking miR-155 are less competent at inducing antigen-specific T cell activation (196). More recently, it was demonstrated that overexpression of miR-155 in DCs is a critical event that is alone sufficient to break self-tolerance in an animal model of diabetes, and promote a CD8-mediated autoimmune response *in vivo* (197). Human CD14^+^ monocytes and macrophages overexpressing miR-155 exhibit increased production of pro-inflammatory cytokines, including IL-1β, IL-6, and TNF, and decreased production of the anti-inflammatory cytokine IL-10 (198).

miR-155-deficient mice display a delayed course and reduced severity of clinical symptoms of EAE (199, 200). Decreased disease severity in miR-155^−/−^ mice was associated with reduced T_H_1 and T_H_17 responses. In addition to the direct effect on T cells, it was also shown that the decreased ability of miR-155 KO mice to mount inflammatory T cell responses was linked to DCs secreting less cytokines critical for driving T_H_1 and T_H_17 responses, mainly IL-1β, IL-6, IL-12, IL-23, and TNF (199). miR-155 is induced in macrophages and DCs after exposure to a variety of inflammatory cytokines such as IFN-β, IFN-γ, and TNF-α (199, 201). It is thus possible to speculate that following the first wave of inflammation, these myeloid APCs upregulate miR-155 leading to an accentuation of the inflammatory response. In addition, more recently, it has been demonstrated that miR-155 plays an essential role in driving the inflammatory phenotype of M1 macrophages (202), which would also impact the severity of the disease. Lastly, treatment with a miR-155 inhibitor after EAE onset reduced the clinical disease severity (199). Considering the important role of miR-155 in driving inflammatory responses in general, and specifically in myeloid APCs, fine tuning the expression of this miRNA in MS would most certainly prompt beneficial results in terms of slowing the inflammatory loop. It is noteworthy that miR-155 is the most consistent miRNA found to be upregulated in MS being reported in eight independent studies (203).

A third miRNA that has been shown to regulate myeloid cell activation is miR-146a. Like miR-155, miR-146a is upregulated following cell stimulation and its induction is NF-κB dependent (204). However, contrary to miR-223 and miR-155, miR-146a represses inflammatory responses by targeting two adapter proteins, TNF receptor–associated factor 6 (TRAF6) and IL-1 receptor-associated kinase 1 (IRAK1), that are crucial for pro-inflammatory signaling (204). miR-146a KO mice develop a spontaneous autoimmune disorder, characterized by splenomegaly, lymphadenopathy, and multiorgan inflammation (205, 206). In addition, miR-146a KO mice display excessive production of myeloid cells and develop flank tumors in their secondary lymphoid organs. Consistent with the repression of inflammation, miR-146a expression promotes M2-Macrophage polarization by targeting Notch-1 (207). Multiple studies have indicated that miR-146a plays pivotal roles in the pathogenesis of several autoimmune diseases, such as systemic lupus erythematosus, rheumatoid arthritis, and Sjögren's syndrome (208). In MS, miR-146a is upregulated in active lesions (179), as well as in PBMCs of RRMS patients (209, 210). Expression of miR-146a is reported to be significantly downregulated in glatiramer acetate treated RRMS patients (210). Logically, upregulation of this miRNA would seem to be beneficial in reducing the ongoing inflammation observed in MS patients leading to the possibility that the upregulation observed in MS patients is the result of the ongoing inflammation rather than a pathological expression. However, when studied in animal models of MS, there was no consensus on the suppressive effects of miR-146a. One study using the Cuprizone-induced demyelination model found that miR-146a-deficient mice displayed reduced inflammatory responses, demyelination, axonal loss, and numbers of infiltrating macrophages compared to WT controls (211). However, a second study found that miR-146a-deficient mice developed more severe EAE characterized by exaggerated T_H_17 responses (212), going along the possible beneficial effect of upregulation of this miRNA in MS. More recently, it was shown that miR-146a mimic treatment of mice with RR-EAE at day 14 improved neurological function, increased the number of newly generated oligodendrocytes, which may facilitate remyelination in the CNS (213). In addition, the treatment increased the number of regulatory M2 macrophages while reducing the number of pro-inflammatory M1 macrophages (213).

Currently, targeting miRNAs is a challenge since they control a myriad of immune and non-immune related functions. However, there is a strong interest in pursuing this approach, not only in MS, but also in many different diseases. Identification of technology to target miRNAs in a cell specific manner would appear to be the desired way to safely and effectively employ this targeting strategy. In the meantime, an abundance of researchers are also exploring the use of miRNAs as biomarkers of diseases pathogenesis and therapy.

## Targeting Immunological Activity of Myeloid Cells

The final strategy we will discuss is the use of nanoparticles to target myeloid cells for disease therapy which has been pioneered in our laboratory. In the recent past, many studies have focused on characterizing the ability of nanoparticles to modulate immune responses and ultimately to be used as potential therapeutics for immune-related diseases. Here we will focus on the “carboxylated” poly(lactic-*co*-glycolic acid) (PLGA) nanoparticles, which are particles without any protein or peptide attached to the surface or encapsulated inside. Phagocytic cells have the extraordinary ability to engulf dead cells, invading microbes and other particles, and this property of phagocyte cells led to the idea of using carriers such as apoptotic cells (214–216), liposomes (217), extracellular vesicles (218), or nanoparticles (219–223) to deliver molecules to modify the immune response. We will restrict our discussion to the use carboxylated PLGA nanoparticles for the modulation of inflammatory monocytes for treatment of CNS inflammation for multiple reasons. Firstly, they can be easily manufactured under GMP conditions. Secondly, they more specifically target inflammatory monocytes by their affinity of binding via the macrophage receptor of collagenous structure (MARCO) (224) as compared to liposomes and extracellular vesicles. Thirdly, they directly carry out immune-modulatory effects on monocytes without the need for add-on agents such as would be required with liposomes. Lastly, they have been proven to be safe and efficacious for use in celiac disease patients treated *via* intravenous infusion of gliadin encapsulating PLGA nanoparticles for induction of immune tolerance in a phase 1/2a clinical trial (225).

Nanoparticles have diameters between 1 and 1,500 nm. Smaller particles (<100 nm) are able to cross tissue barriers and traffic directly to the lymph nodes. Larger particles (>100 nm) require uptake by phagocytic cells (226). Nanoparticles administered subcutaneously or intradermally may be taken up by tissue resident APCs or their precursor cells and are ultimately transported to the draining lymph nodes. Systemic administration of nanoparticles favors accumulation in the organs such as the spleen and liver (227). Also, the shape of nanoparticles dictates efficiency of uptake by phagocyte cells. For example, phagocyte cells internalize spherical-shaped nanoparticles more easily than stretched-shaped structures (228). And although positively charged particles are taken up more avidly, negatively charged particles have been shown to exhibit lower toxicity (229–232). Nanoparticles can be made from different materials, metallic (e.g., silver, gold, and copper), magnetic (e.g., iron) (useful for imaging), ceramic, carbon-based, silica, lipid-based, or polymeric such as poly(amino acids), polysaccharides and poly(alpha-hydroxy acids).

Our group was one of the first to test the ability of 500 nm non-biodegradable carboxylated polystyrene (PS) particles to modulate immune responses in inflammatory settings *in vivo*. Intravenous infusion of PS nanoparticles led to a reduction in trafficking of Ly6C^hi^ inflammatory monocyte into the CNS and increased survival in a mouse model of West Nile virus (WNV) encephalitis (224). It was discovered that these inflammatory monocytes were redirected to the spleen of treated animals and resulted in a dramatic reduction of mortality in WNV-infected mice by preventing the release of a pro-inflammatory “cytokine storm” in the CNS. Robust anti-inflammatory effects induced by infusion of PS nanoparticles were also observed in other inflammatory diseases such as peritoneal inflammation and inflammatory bowel disease. To enhance the clinical relevance of the nanoparticle targeting approach, we next investigated the potential of biodegradable carboxylated PLGA nanoparticles for regulation of myeloid cell-dependent inflammation.

PLGA is one of the best characterized and most used biodegradable polymers. The hydrolysis of PLGA leads to metabolite monomers, lactic acid and glycolic acid. The two monomers are endogenous and easily metabolized by the body via the Krebs cycle. There is minimal systemic toxicity associated with the use of PLGA (233, 234). Because PLGA is a safe material, it has been approved by the United States Food and Drug Administration (FDA) and European Medicine Agency (EMA) in various drug delivery systems in humans. Indeed, PLGA can be engineered to deliver, alone or in any combination with small-molecule drugs, proteins, peptides, DNA, miRNAs, and even clustered regularly interspaced short palindromic repeat (CRISPR) (227).

We have shown that administration of negatively charged 500 nm PLGA nanoparticles resulted in reduced inflammatory monocytes accumulation and overall robust beneficial effects in disease severity in multiple mouse models of inflammatory disease such as EAE (224), SCI (235), TBI (236), myocardial infarction (237), and herpes simplex virus 1 infection of the cornea (238). The exact mechanisms behind immunomodulatory effects of PLGA therapy are still under investigation. However, in all these models, it has been shown PLGA particles are selectively recognized and bound by inflammatory monocytes. These monocytes undergo sequestration and eventual apoptosis in the spleen, culminating in reduced immune pathology at sites of inflammation. Phenotypic changes were also observed on DCs and macrophages in the inflammatory sites, showing decreased expression of activation markers such as MHC II and CD86. In the SCI study, PLGA nanoparticle administration led to reduced M1 macrophage polarization.

While our group has also shown that antigen (Ag)-coupled or encapsulated PLGA nanoparticles can have important immunomodulatory effects (220, 222, 224, 239), other strategies using PLGA nanoparticles have also been shown to regulate EAE (240). For example, Cappellano et al. showed that simultaneous subcutaneous injection of PLGA nanoparticles loaded with either MOG_35−55_ or IL-10 ameliorated the course of EAE (241). TGF-β, another immunoregulatory molecule, coupled to the surface of PLGA nanaopartlces containing PLP_139−151_ peptide were shown to improve the tolerogenic effect of Ag-PLGA nanoparticles (242). Another example is Maldonaldo et al. using PLGA nanoparticles loaded PLP_139−151_ together with rapamycin, an inhibitor of the mTOR pathway, and demonstrating that a single dose of these particles injected at the peak of disease were able to protect from relapses (243). Also, Pei et al. aimed to develop PLGA nanoparticles which function as a direct modulator of T cells, without the involvement of APCs (244). For that purpose, TGF-β1 encapsulated nanoparticles were coupled with target antigens for CD4 and CD8 T cells (MOG_40−54_/H-2D^b^-Ig dimer and MOG_35−55_/I-A^b^ multimer), regulatory molecules (anti-Fas and PD-L1-Fc) and a “self-marker” CD47-Fc (244). These particles were injected in EAE mice on day 8, 18, 28, and 38 after immunization with MOG_35−55_, and induced a significant reduction in EAE symptoms that lasted for more than 100 days. Moreover, the authors observed a decrease of T_H_1 and T_H_17 MOG_35−55_-specific cells as well as T_C_1 and T_C_17 MOG_40−55_-specific cells, an increase of regulatory T cells, inhibition of T cell proliferation and augmentation of T cell apoptosis in the spleen (244). In addition to regulating the immune response, PLGA nanoparticles have also been used as a transporter to help in the remyelination process. Indeed, Rittchen et al. encapsulated leukemia inhibitory factor (LIF), which is a cytokine known to promote oligodendrocyte maturation thus favoring remyelination (245). To specifically target oligodendrocytes, the LIF-PLGA nanoparticles were coupled with anti-NG2 antibodies. The authors showed that intra-lesion delivery of LIF-PLGA nanoparticles improved CNS remyelination increasing the percentage of remyelinated axons and their thickness (245).

In conclusion, the mechanism(s) of action of PLGA nanoparticles are still incompletely understood, but studies in multiple models have shown their capacity to limit inflammatory events by targeting inflammatory monocytes. PLGA nanoparticles can also be used as delivery vectors, like liposomes and extracellular vesicles. However, a critical advantage of carboxylated PLGA nanoparticles, as compared to liposomes and extracellular vesicles, is their ability to act directly to modulate the function and trafficking of inflammatory monocytes based on their ability to engage the MARCO scavenger receptor. Because of the safety record of PLGA nanoparticles, they can be easily translated into clinical use. In fact, Cour Pharmaceuticals successfully completed a Phase IIa clinical trial for celiac disease showing the safety and efficacy of systemic infusion of PLGA nanoparticles encapsulating gliadin for inducing gluten-specific immune tolerance in celiac disease patients undergoing oral gluten challenge. Takeda Pharmaceuticals has acquired the exclusive license for future development of this therapy for celiac and other GI diseases. Cour Pharmaceuticals is currently developing antigen encapsulating PLGA nanoparticle-based tolerance clinical programs for treatment of MS and peanut allergy and clinical programs using carboxylated “naked” PLGA nanoparticles targeting inflammatory monocytes for treatment of acute respiratory distress in COVID-19 infection and treatment of TBI.

## Conclusions

**Figure 1:**
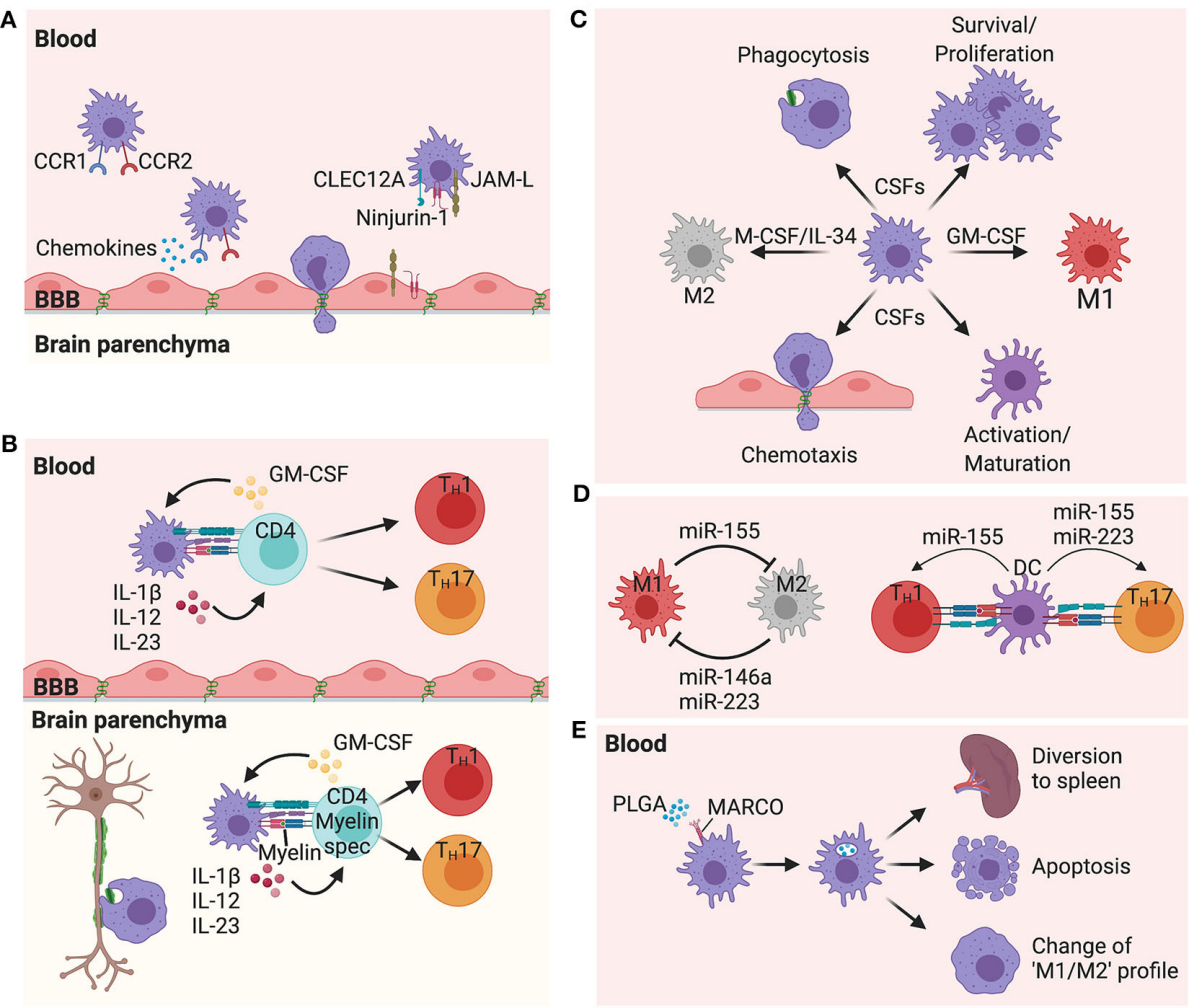
Proposed mechanisms of peripheral myeloid cells in multiple sclerosis pathogenesis. **(A)** Chemokine receptors CCR1 and CCR2, as well as the adhesion molecules Ninjurin-1 and JAM-L, and the C-lectin receptor CLEC12A have all been shown to play important roles in myeloid cell migration from the periphery to the CNS. **(B)** Interactions between myeloid cells and CD4 T cells are critical to shape the type of immune response. These interactions take place in the periphery as well as in the CNS. Cytokines such as IL-1β, IL-12, and IL-23 produced by myeloid cells and GM-CSF produced by activated CD4 T cells are all attractive targets for MS therapy, playing critical roles in the CNS inflammation. **(C)** M-CSF (CSF-1)/IL-34 and GM-CSF (CSF-2) are upregulated during inflammation and control many different functions on myeloid cells such as differentiation, phagocytosis, chemotaxis, activation, polarization, and survival. Blockade of the receptors of these growth factors are intriguing therapeutic options in MS. **(D)** miRNAs have the ability to regulate the function of many cells, including myeloid cells. miR-223,−155, and−146a have all shown to be upregulated in MS lesions, and are expressed by myeloid cells. While miR-146a and miR-223 promote an M2 profile, miR-155 promotes an M1 profile from macrophages/microglia. However, expression of miR-223 and miR-155 by DCs induce the ability of these cells to promote a T_H_17 polarization. miR-155 also has the ability to promote a T_H_1 polarization when expressed by DCs. **(E)** Intravenous injection of PLGA nanoparticles are capture by MARCO+ myeloid cells leading these cells to be sequestered in the spleen, with some cells dying by apoptosis and other cells changing their profile toward an immunoregulatory phenotype.

The importance of peripheral myeloid cells in MS pathology is profound. There is an extensive presence of these cells and their products in MS lesions as well as in the CSF of MS patients. Studies in animal models of MS have clearly demonstrated the beneficial effects in targeting peripheral myeloid cells for the different forms of the disease. Multiple tools have now been developed targeting these cells including blockade of their migration to the CNS, their activation and cytokine production, their biological functions and their immunological activity (Figure 1). However, contrary to other inflammatory disorders, no drug is currently approved targeting specifically these cells in MS. Multiple pro-inflammatory cytokines including GM-CSF, IL-1β, IL-12, IL-23, M-CSF all represent potential MS therapeutic targets. Treatments targeting these cytokines have been shown to be well-tolerated and safe in patients for different diseases. Additionally, non-specific blockade of leukocyte entry to CNS using Natalizumab is beneficial in MS, however this carries the risk of severe side-effects from infections. However, specifically impeding the migration of myeloid cells would limit such adverse effects. CLEC12A, CCR1, CCR2, JAM-L, and Ninjurin-1 represent interesting options to inhibit CNS migration of peripheral myeloid cells. Altering the biological functions of myeloid cells via through miRNA modulation is an appealing strategy for treating MS and other chronic inflammatory diseases. miR-146a, miR-155, and miR-223 are all upregulated on myeloid cells from MS patients. Lastly, nanoparticles represent one of the most exciting new tools for regulating myeloid cell functions. The biodegradable PLGA particles are particularly interesting due to their approval by the FDA and EMA for use in humans, as well as their ability to regulate many different inflammatory disorders, even those that take place in the CNS.

## Author Contributions

II and SM provided intellectual contribution to the work. II wrote the manuscript. SM provided guidance, edited, and reviewed the manuscript. Both authors approved the manuscript for publication.

## Conflict of Interest

SM is a cofounder of Cour Pharmaceutical Development Co and also a member of the scientific advisory board and consultant for Cour Pharmaceutical Development Co as well as a shareholder. The remaining author declares that the research was conducted in the absence of any commercial or financial relationships that could be construed as a potential conflict of interest.
